# 

**Published:** 1883-07

**Authors:** 


					﻿L.SURG GENlS OFFS
Voi.-xxr
July, 1883.
No. 2.
ThE
1ST0URY.
IT-wk /Tfr	>£'jjag KS
eR™ i SfflW ifllSfc
____	w i | W n ?
ELMIRA.N.Y.
				

